# Internet-Delivered Acceptance and Commitment Therapy Added to Multimodal Pain Rehabilitation: A Cluster Randomized Controlled Trial

**DOI:** 10.3390/jcm10245872

**Published:** 2021-12-14

**Authors:** Nina Bendelin, Björn Gerdle, Marie Blom, Martin Södermark, Gerhard Andersson

**Affiliations:** 1 Pain and Rehabilitation Centre, Department of Health, Medicine and Caring Sciences, Linköping University, 581 83 Linköping, Sweden; bjorn.gerdle@liu.se (B.G.); marie.blom@gmail.se (M.B.); martin.sodermark@liu.se (M.S.); 2 Department of Behavioural Sciences and Learning, Linköping University, 581 83 Linköping, Sweden; gerhard.andersson@liu.se; 3 Department of Biomedical and Clinical Sciences, Linköping University, 581 83 Linköping, Sweden; 4 Department of Clinical Neuroscience, Karolinska Institute, 171 77 Stockholm, Sweden

**Keywords:** chronic pain, internet-delivered acceptance and commitment therapy (IACT), multimodal pain rehabilitation program (MMRP), aftercare, implementation, internet-delivered cognitive behavioral therapy (ICBT), booster intervention, combined treatment

## Abstract

Internet-delivered interventions hold the possibility to make pain rehabilitation more accessible and adaptable by providing qualified individualized psychological care to chronic pain patients in their homes. Acceptance and commitment therapy (ACT) has shown promising results on psychological functioning and pain acceptance. Internet-delivered ACT (IACT) added to multimodal pain rehabilitation program (MMRP) in primary care has, so far, not shown better results than MMRP alone. The aim of this cluster randomized controlled study was to investigate the effects of adding IACT during and after MMRP in specialist care on psychological outcomes. In total, 122 patients who enrolled in a specialist pain clinic were cluster randomized groupwise to either MMRP (*n* = 12 groups) or to MMRP with added IACT (*n* = 12 groups). The IACT addition included 6 weeks of treatment during MMRP and 11 weeks of aftercare following MMRP. Online and paper-and-pencil self-report measures of pain acceptance, psychological inflexibility, self-efficacy, and psychosocial consequences of pain, were collected at four occasions: prior to and post MMRP, post aftercare intervention and at 1 year follow-up. Dropout was extensive with 25% dropping out at post treatment, an additional 35% at post aftercare, and 29% at 1 year follow-up. Medium treatment between-group effects were found on pain acceptance in favor of the group who received IACT added to MMRP, at post treatment and at post aftercare. Large effects were seen on psychological inflexibility and self-efficacy at post aftercare. A medium effect size was seen on affective distress at post aftercare. Moreover, a medium effect on self-efficacy was found at 1 year follow-up. The results indicate that IACT added during MMRP may enhance the treatment effects on pain-related psychological outcomes. Results also suggest that IACT as aftercare may strengthen the long-term effect of MMRP. However, adding a second pain treatment, IACT, to an already extensive pain treatment, MMRP, could be perceived as too comprehensive and might hence influence completion negatively. Further research on adverse events and negative effects could be helpful to improve adherence. Next step of implementation trials could focus on adding IACT before MMRP to improve psychological functioning and after MMRP to prolong its effect.

## 1. Introduction

The need for specialist care for chronic pain patients is extensive and expanding [1]. Multimodal pain rehabilitation programs (MMRP) are bio-psycho-social treatments focusing on return to work and improved physical and psychological functioning [2]. These interdisciplinary treatments are conducted in primary care and in specialist clinics for patients with pain and mild to moderate psychiatric comorbidity. The composition of team members and interventions, and the length and extensiveness of the programs may vary. They are, however, often based on cognitive behavioral therapy and combine psychological interventions as behavioral activation with guided physical activity and educational sessions and skills training.

Although specialist care MMRPs are regarded as evidence-based treatments with small but significant outcomes, and are well-integrated in clinical services [3], they possess some concerns. Aftercare for this chronic condition often is unregulated, varies in content and length and may either be part of MMRP or managed in primary care settings [4,5]. In addition, accessibility may be limited due to geographical distances [6], financial restrictions [7] or mobility difficulties [8].

Internet-delivered interventions enable chronic pain patients to access qualified care in their homes [9] and may possibly support self-help after rehabilitation [10]. Home-based rehabilitation extends the care of the professionals [6] and enables repeated training with support from a social network at a times convenient for the individual [11]. Internet-delivered interventions may stimulate self-management as well as raise attendance and engagement due to their flexibility, anonymity, and emphasis on autonomy [8].

Effectiveness studies suggest that internet-delivered psychological interventions may function on their own or as a part of stepped care in regular health services [11]. Internet-delivered acceptance and commitment therapy (IACT) has shown promising results for patients with chronic pain, although it is still an under-researched area [9]. ACT builds on cognitive behavioral therapy (CBT) and includes methods for experiential learning. ACT aims to help patients behave in a more psychologically flexible manner towards feelings, thoughts and bodily sensations, by fostering acceptance, defusion and awareness, and committing to actions in line with individual values [12]. It has been listed as an empirically supported treatment for chronic pain [13] and has, as CBT, been successfully transferred to internet delivery mode [9]. Thus, so far, IACT for chronic pain has shown small to large effect sizes on pain-related outcomes as pain interference, pain intensity and disability, and on psychological outcomes as acceptance, anxiety, depression, catastrophizing and fear-avoidance [14].

With regards to specialist care specifically, a non-randomized trial of IACT (*n* = 99) with individualized coaching given as a stand-alone treatment, showed improvements on self-efficacy, activity engagement, pain intensity, pain interference and treatment satisfaction [15]. An uncontrolled trial of IACT provided in a tertiary care setting (*n* = 39) showed significant improvements on depression, anxiety and disability, for a subgroup of participants [16]. In addition, a wait-list control trial on IACT for chronic pain patients in tertiary care (*n* = 113), although self-referred, showed treatment effects on pain interference, depression, anxiety, pain intensity and insomnia [17]. Finally, IACT as standalone treatment given to patients recruited from a tertiary care center (*n* = 33), although without an active comparison, showed significant improvements on depression and pain intensity [18].

Internet-delivered interventions combined with face-to-face treatment may be promising in behavior change treatments for chronic somatic disorders, as the setup enables patients to take an active role in self-management [6]. The most effective composition is still unclear [6,8]. However, booster sessions, a multiple behavior approach and multimedia, have been suggested as important parts [6,19].

Internet-delivered interventions added to MMRP are however sparsely studied. One reason might be that it can be ethically problematic to withhold patients MMRP while testing an internet-delivered intervention. Another reason might be that trials in clinical settings may encounter obstacles due to the vast symptom panorama of chronic pain patients as well as social factors that affect adherence and hence lead to missing data [11,20,21]. In a study where ICBT was added during MMRP in primary care (*n =* 109), there was an effect on catastrophic thinking and on treatment satisfaction [22]. However, this study yielded no treatment effects on self-efficacy, pain intensity, coping strategies [22], work ability, disability and health-related quality of life [23].

To summarize, IACT may be helpful for chronic pain patients as a stand-alone treatment [9], as part of stepwise care [11], or as a complement to face-to-face treatment [24]. However, the potential contribution of adding IACT during or after MMRP in a specialist care setting is less researched.

The aim of this randomized controlled trial was to investigate the effect of IACT added to MMRP (here after called MMRP-IACT) for chronic pain patients in specialist care, on pain-related psychological outcomes, as pain acceptance, psychological flexibility, self-efficacy, and affective distress.

## 2. Materials and Methods

### 2.1. Design

The study was a 2-year cluster-randomized controlled trial with two intervention arms: MMRP and MMRP with an IACT addition. The trial had two phases. First, IACT added during MMRP was compared against MMRP alone for six weeks. Thereafter, IACT as aftercare was compared to aftercare as usual following MMRP for 11 weeks. Measurements were collected prior and post MMRP, post aftercare intervention and 1 year after end of MMRP. Follow-up time was set to 1 year to be consistent with data collection through the Swedish Quality Registry for Pain Rehabilitation (SQRP). The study protocol was retrospectively registered at ClinicalTrials.gov, accessed on 1 June 2021 (NCT05071547).

### 2.2. Recruitment and Participants

During an inclusion period of 2 years, all 258 patients scheduled for MMRP at a specialist pain clinic, i.e., the Pain- and Rehabilitation Centre, University Hospital, Linköping, Sweden, were consecutively assessed for enrollment. To be included, patients had to (1) meet the inclusion criteria for MMRP (chronic pain with duration >6 months with mild to moderate psychiatric symptoms assessed by pain specialist physician at enrollment at the specialist pain clinic, using data from SQRP) and (2) receive the allocated face-to-face MMRP including ACT group sessions. Exclusion criteria were (1) <18 years of age, (2) difficulties reading and writing in Swedish, (3) unable to work at a computer. Participants’ characteristics data were collected at enrollment. Participants were financially compensated for the effort of completing follow-up measures. Recruitment started November 2010 and ended December 2012.

Most participants were women (85.4%, *n* = 88). The mean age at enrollment was 36.13 years (SD 9.68) and the mean pain duration was 7.1 years (SD 7.0). Mean rating of pain intensity last week, VAS (visual analog scale ranging from 0 “no pain” to 10 “worst pain imaginable”) was 7.05 (SD 1.64). A total of 36.9% (*n* = 38) received sickness benefits to any degree. The highest educational attainment was elementary school for 8.7% (*n* = 9) of the participants, secondary school for 58.3% (*n* = 60) and university for 23.3% (*n* = 24) (Table 1). At enrollment, 86.2% (*n* = 75) of the participants reported prescribed pain medication during the two previous weeks. Mental health medication was reported by 23.0 % (*n* = 20) and 57.5% (*n* = 50) reported medication for other somatic conditions. Participants had different common chronic pain conditions, e.g., widespread pain including fibromyalgia, low back pain, neck-shoulder pain, etc.

Data on participants’ characteristics were collected through SQRP. Data on medication use were measured using a self-report form, Treatment Inventory Cost in Psychiatric patients (TIC-P) [25]. TIC-P is an instrument to collect information on societal and health care costs relating to psychiatric illness for economic evaluations [26]. No statistical differences between the groups (i.e., MMRP-IACT vs. MMRP) were found for age, pain severity, pain duration, working condition, sick-leave, financial compensation, or educational attainment (Table 1).

### 2.3. Randomization

All patients enrolled in MMRP at the clinic during the study time were cluster randomized in their respective clinical rehabilitation group prior to inclusion, to either (MMRP, *n* = 12 groups) or MMRP with an internet-delivered addition (MMRP-IACT, *n* = 12 groups). An online true random-number service (www.random.org, (accessed on 1 November 2010)) was used by a research assistant not otherwise involved in the trial, who informed the research team shortly before start of each MMRP group about group allocation. There were no statistically significant differences (*p* > 0.05) between the randomized clusters on pre-treatment data.

The randomization procedure was an inevitable consequence of implementation in clinical practice, to avoid participants in the same MMRP group being allocated to different treatment groups. Even though cluster randomization is less optimal compared to randomization on an individual level, this design was still chosen for ethical reasons. To respect the integrity of the MMRP groups we did not want to risk disturbing the group processes in MMRP groups or the positive influence of patients sharing their experiences of treatment. It was, however, possible for individual participants to decline participation while other group members stayed included. 

### 2.4. Procedure

When patients consented to participate, additional written information was given and followed up either over telephone or at a clinical visit as they were introduced to the application. The research team was blind to the randomization until shortly prior to start of each MMRP group. The clinical staff in MMRP were not blind to allocation. Data were primarily collected digitally. The complementary data from SQRP was administered by a research assistant blind to allocation. 

After completing pre-measurements, participants were assigned to either the intervention module (MMRP-IACT) or the control group module (MMRP). A total of 122 participants consented to participate in the study, MMRP (*n =* 61) or MMRP-IACT (*n* = 61). Of these, 103 participants filled in pre-measurements and were included in the study (MMRP-IACT, *n* = 49; MMRP, *n* = 54).

**Figure 1:**
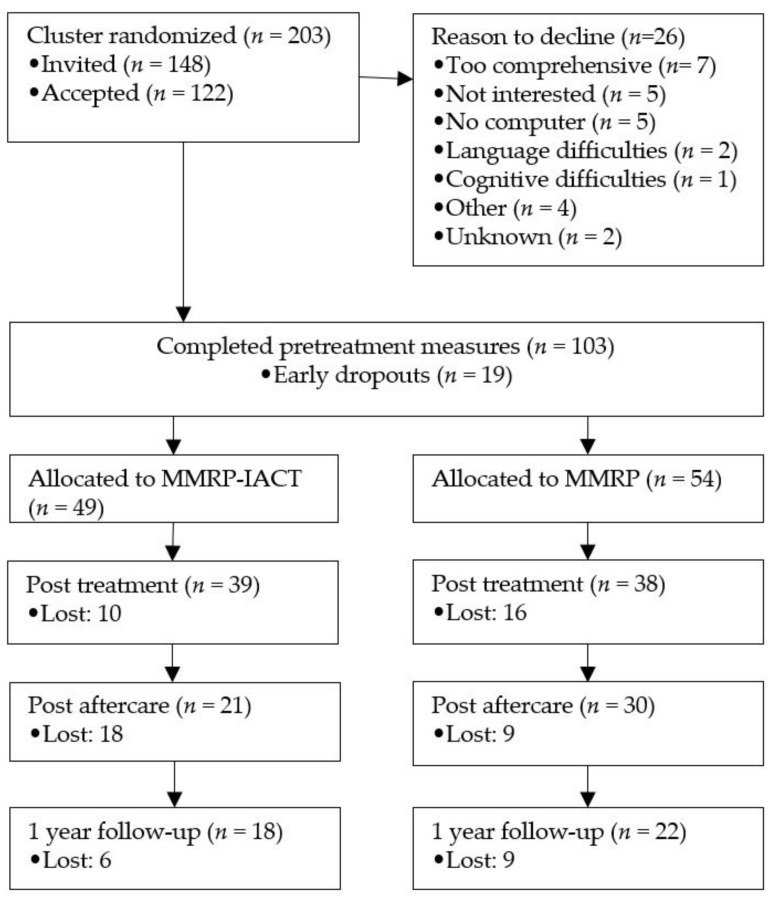
Flowchart.

In total, *n* = 55 were assessed as not eligible for inclusion in the study. The main reason (*n* = 50) was that they had been scheduled for individual pain rehabilitation rather than group-based MMRP when they were admitted to the pain clinic. The 26 patients who declined participation (Figure 1) received MMRP as usual, although not associated to the study. Early dropouts were defined as persons who either actively declared non-interest after having accepted participation and filled in pre-measurements or did not fill in pre-measurements.

### 2.5. Interventions

#### 2.5.1. MMRP

The 6-week long group-based MMRP included approximately 108 h of 60–120 min long treatment sessions on site and focused on return to work [3]. Patients attended the MMRP four days a week, approximately 5.5 h from 8.30 a.m. to 14.00 p.m. One day a week was reserved for home-based activities. Psychologists, physicians, physiotherapists (PT) and occupational therapists (OT) gave synchronized treatments with a CBT/ACT approach and collaborated extensively during assessment, individual treatment planning and continuous process evaluation, by IASP defined as interdisciplinary treatment [27]. PT sessions consisted of four kinds of physical training; basic body awareness, circuit training, light aerobic and beginner workout, as well as educational interventions, and home-based low-intensive physical exercise started prior to MMRP. OT sessions had an overall focus on return to work, although also covering a variety of interventions promoting activity pattern and -execution, and occupational role and -significance. ACT sessions targeted experiential avoidance with interventions from original ACT literature and modified to chronic pain patients [28,29,30]. The insomnia intervention [31] included sleep restriction, stimulus control, sleep hygiene, worry and relaxation, and was slightly altered to be congruent with the ACT-sessions. The MMRP also included educational sessions [3] and individual sessions upon request (see Supplementary Materials Table S1 for outline of content in MMRP). After end of MMRP, the majority of patients we routinely re-referred to their respective primary care center, without aftercare.

#### 2.5.2. MMRP-IACT

Participants allocated to the MMRP-IACT condition participated in face-to-face MMRP as described above. In addition, they received individual IACT adjusted to fit with MMRP. The IACT addition supplied participants with weekly educational material and additional exercises in line with face-to-face MMRP, although enriched with multimedia. This way, participants had access to rehabilitation via the website and could practice in their homes in-between face-to-face sessions of MMRP. Educational texts were approximately half an A4 page, focused on a theme, presented therapeutic exercises, mindfulness exercises and guiding questions to help participants apply the theme [28,29,32,33]. There were also interactive work sheets for homework administration, a physical exercise diary, and extra clarifying educational texts and exercises. The chapters were brief, comprehensive, and easy to read or listen to for approximately 15 min. Some parts, for example mindfulness exercises were meant to be listened to repeatedly. (See outline of IACT added during MMRP in Supplementary Materials Table S2). Participants were encouraged to evaluate their progress using the Bull’s-eye values survey (BEVS) [34], an illustrated figure known from face-to-face ACT sessions. An e-therapist (psychologist trained in ACT) gave feedback on exercises and homework and was available online for questions. The feedback focused on continuous practice, problem-solving and staying goal-focused. Although new material was posted once a week, participants were encouraged to log on repeatedly throughout the week to report homework assignments, read e-therapist feedback and practice mindfulness.

Phase 1 (IACT during MMRP) ended simultaneously as the MMRP. A pause of 1 month was set to collect post MMRP data and for patients to reengage in work and studies, in line with the usual procedure after MMRP. Participants were thereafter invited to Phase 2 of the study (IACT after MMRP); an 11-week long aftercare program, focusing on maintaining progress and generalizing skills to home and work settings. Besides a few mandatory modules, participants were encouraged to choose from optional modules corresponding with residual symptoms or areas they had previously neglected (see outline of IACT added after MMRP in Supplementary Materials Table S3).

The technical solution behind the plat form used for delivering the IACT intervention was designed and developed for the present trial. It was equivalent to recent plat forms, with multimedia components and synchronic communication. E-therapists guided patients and could edit the material. The delivery format was text, audio, and videos. The plat form was patient-interactive and the content was partly tailored to the needs of the patient. The content enabled a combination of internet-delivered and site based face-to-face treatment and the interface was accessible through different tablets. The IACT program was validated by two experts who have long clinical and research experience from internet-delivered psychological interventions (GA) and pain rehabilitation (BG).

### 2.6. Outcome Measures

Outcome variables were mainly chosen according to recommendations in IMMPACT guidelines [35,36] and collected digitally on 4 occasions; at pre and post treatment, at post aftercare intervention and at 1 year follow up. Additional complementary pen-and-paper data were drawn from the SQRP at three occasions; at pre-baseline/enrollment, at post treatment and at 1 year follow up. Outcome measures included psychological outcomes: pain acceptance, psychological inflexibility, self-efficacy, and psychosocial consequences of living with pain.

#### 2.6.1. The Chronic Pain Acceptance Questionnaire (CPAQ)

CPAQ is a measure of pain acceptance with 20 items rated on a 6-point scale ranging from “never true” to “always true” and divided into two subscales: activity engagement and pain willingness [37,38]. CPAQ has been validated for a Swedish sample [38] and for an internet sample [39]. Studies have shown high test-retest reliability (α = 0.72–0.92). The internal consistency in the present sample at pre-measurement was high (α = 0.87).

#### 2.6.2. Psychological Inflexibility in Pain Scale (PIPS)

Twelve items are rated on a 7-point scale ranging from “never true” to “always true” and divided into two subscales: avoidance and cognitive fusion [40]. Higher score means higher psychological inflexibility. PIPS has been found a valid and reliable measure which may function as a working mechanism in ACT for chronic pain [41]. The internal consistency in the present sample at pre-measurement was high (α = 0.91).

#### 2.6.3. Pain Self-Efficacy Questionnaire (PSEQ)

PSEQ is a 10-items measure of chronic pain patients’ perception of their ability to cope with discomfort. The 7-point scale ranges from “not sure at all” to “entirely sure” [42]. Higher score means better perceived self-efficacy. Test-retest reliability has been found high [43]. The internal consistency in the present sample at pre-measurement was high (α = 0.89).

#### 2.6.4. Multidimensional Pain Inventory (MPI)

The West Haven–Yale Multidimensional Pain Inventory (MPI) is a measure of psychosocial, cognitive, and behavioral components relating to chronic pain [44,45,46]. MPI is divided into three parts. Part 1 of the Swedish version [47] was used in this trial. It consists of 28 items rated on a 7-point scale and divided into five subscales with good reliability (pain severity *α* = 0.75, pain interference *α* = 0.85, life control *α* = 0.81, affective distress *α* = 0.74 and social support *α* = 0.88 [44,48]. The internal consistency in the present sample at pre-measurement was acceptable or high, pain severity *α* = 0.80, pain interference *α* = 0.85, life control *α* = 0.67, affective distress *α* = 0.77 and social support *α* = 0.83.

### 2.7. Statistical Analyses

The IBM SPSS version 26.0 (IBM Corporation, Route 100 Somers, New York, NY, USA) was used for descriptive statistics, Mann–Whitney U-test, Student’s *t*-test, and Pearson’s Chi-squared test; *p* < 0.05 was considered significant in all statistical tests. As an active comparator was used as the control condition, an effect size of *d* = 0.30 (specific component comparator [49]) was expected. Given 80% power and a 5% significance level, the sample size calculation indicated that 90 participants in each group were needed. Considering a 50% dropout rate [50,51], a sample size of 135 participants in each group would be sufficient. We planned to include *n* = 300. Due to problems with recruiting and missing data, the study included substantially less than 300 complete cases. Effect sizes for paired observations were calculated using a web-calculator [52]. Effect sizes were considered small if 0.20–0.49, medium if 0.50–0.79 and large if >0.80.

Variances were large due to outliers and data were not found to be normally distributed. Hence, parametric tests were not used. As regression slopes were heterogenous, one assumption for ANCOVA was violated. Due to large number of missing in a relatively small sample, mixed models were found less optimal as the data were not considered robust enough [53]. In addition, Little’s MCAR test indicated that the missing data were not random. We reached the conclusion to perform non-parametric tests using Mann–Whitney U-test for pair-wise comparisons. Independent samples *t*-test complete cases analysis was used as complementary control. As missing data were too extensive to ignore, an intention to treat analysis was not motivated and we decided not to impute for missing data [53]. All eligible data were included in the analyses.

## 3. Results

The trial investigated the effect of adding IACT during and after an existing MMRP in specialist pain care. Descriptive statistics over time are reported in Table 2. In conclusion, there was a statistically significant effect in favor of the MMRP-IACT group on pain acceptance (CPAQ total scale and pain willingness subscale) post treatment and post aftercare. There was also a statistically significant effect in favor of the MMRP-IACT group on psychological inflexibility (PIPS total scale, the avoidance subscale, and the fusion subscale) post aftercare. A statistically significant effect was found on both affective distress (MPI subscale) and on pain-specific self-efficacy (PSEQ) post aftercare. Pain-specific self-efficacy (PSEQ), but no other outcomes, differed statistically significant between the groups at 1 year follow-up. 

### 3.1. Pain Acceptance

There was a statistically significant difference between the groups on CPAQ total scale at post treatment (Mann–Whitney U = 493.5, n1 = 37, n2 = 37, *p* = 0.039), with a medium effect size (*d* = 0.50, CI 95%: 0.04, 0.96). There was also a statistically significant difference on the CPAQ pain willingness subscale at post treatment (Mann–Whitney U = 475.5, n1 = 39, n2 = 37, *p* = 0.01), with a medium effect size (*d* = 0.60, CI 95%: 0.14, 1.06).

At post aftercare, there was a statistically significant difference on CPAQ total scale (Mann–Whitney U = 182.5, n1 = 19, n2 = 30, *p* = 0.035), with a medium effect size (*d* = 0.63, CI 95%: 0.04, 1.22). Likewise, the CPAQ pain willingness subscale differed statistically significant (Mann–Whitney U = 185, n1 = 19, n2 = 30, *p* = 0.04) at post aftercare, with a medium effect size (*d* = 0.61, CI 95%: 0.02, 1.20).

### 3.2. Psychological Inflexibility

There was a statistically significant difference between the groups on PIPS total scale at post aftercare (Mann–Whitney U = 193, n1 = 21, n2 = 30, *p* = 0.019), with a large effect size (*d* = 0.96, CI 95%: 0.37, 1.55). The two groups also differed statistically significant on the PIPS avoidance subscale post aftercare (Mann–Whitney U = 206, *p* = 0.037), with a large effect size (*d* = 0.89, CI 95%: 0.31, 1.47). Likewise, there was a statistically significant difference on the PIPS fusion subscale post aftercare (Mann–Whitney U = 189, *p* = 0.016), with a large effect size (*d* = 0.98, CI 95%: 0.39, 1.57).

### 3.3. Affective Distress

There was a statistically significant difference between the groups on the MPI subscale affective distress at post aftercare (Mann–Whitney U = 512, n1 = 41, n2 = 37, *p* = 0.013), with a medium effect size (*d* = 0.58, CI 95%: 0.13, 1.03). There were no statistically significant differences between the two groups on the MPI subscales pain severity, pain interference, life control and social support.

### 3.4. Pain Self-Efficacy

There was a statistically significant difference on PSEQ at post aftercare (Mann–Whitney U = 173.5, n1 = 21, n2 = 30, *p* = 0.007), with a large effect size (*d* = 0.82, CI 95%: 0.24, 1.40). In addition, at 1 year follow up, there was a statistically significant difference on PSEQ (Mann–Whiney U = 125, n1 = 18, n2 = 22, *p* = 0.047), with a medium effect size (*d* = 0.66, CI 95%: 0.02, 1.30).

### 3.5. Attrition

Data lost to follow up were extensive although it did not differ statistically significant between the MMRP-IACT group and the MMRP group, neither at post treatment, at post aftercare nor at 1 year follow-up. In total, 25% (*n* = 26) were lost to post treatment. Of the remaining, a total of 35% (*n* = 27) were lost to post aftercare. Another 29% (*n* = 15) were lost to 1 year follow up. Reasons for attrition were not reported. The numbers of missing do not match the number of respondents at all time points (see Figure 1), as some respondents missed one measurement but did complete a later one. In total, only 39 % (*n* = 40) of the allocated participants (*n* = 103) completed 1 year follow-up.

In the MMRP-IACT group, 20 % (*n* = 10) were lost to post treatment. In the MMRP group, 30 % (*n* = 16) were lost to post treatment. There were no significant differences between completers and non-completers in the intervention group, regarding gender, age, pain severity, educational attainment, reported medication use, occupational degree of sick-leave compensation at baseline. There was however a statistically significant difference between completers and non-completers within the MMRP-IACT group on PIPS total scale at baseline (Mann–Whitney *U* = 284, n1 = 39, n2 = 10, *p* = 0.026).

### 3.6. Delivery of Intervention

Participants completed on average 18.39 chapters (SD 10.23) of IACT during MMRP, ranging from 3 to 59. During the aftercare part, 33 % of participants completed more than the mandatory parts, meaning that they chose optional modules based on individual goals. Participants in the aftercare part completed on average 10.67 chapters (SD 8.77), of IACT during the aftercare part, ranging from 1 to 27.

## 4. Discussion

### 4.1. Principal Findings

This study investigated the effects of adding IACT during and after MMRP for chronic pain patients in specialist care. Treatment effects with medium to large effect sizes were seen on pain acceptance, psychological inflexibility, affective distress, and self-efficacy. Effect sizes are in line with the expected range when comparing with an equivalent condition (specific factors component d = 0.35; 95% CI: 0.11–0.58) [49]. Treatment effects are also in line with effect sizes found in a systematic review of internet-delivered psychological therapies although on disability and pain measures, compared to mostly WLC [24]. However, large missing data calls for cautious interpretations of the result.

An umbrella review of effectiveness of MMRP showed that psychological outcome measures constituted 19% of reported outcome in meta analyses of chronic pain, compared to pain related outcomes (30%), work related (25%) and disability measures (23%) [54]. In this study, we focused on the potential effect of an IACT addition. Hence, we measured psychological outcomes, such as pain acceptance and psychological inflexibility. Although both of these are ACT-oriented treatment components, they differ as pain acceptance is closer associated with a person’s intentions and actions in relation to pain, meanwhile psychological flexibility refers to a person’s ability to govern actions towards desired goals in the presence of pain, emotions and thoughts [12]. Psychological flexibility is also defined as the desirable long-term effect of ACT [55]. Emotional distress was chosen to measure the psychosocial impact of pain. Our interest in self-efficacy was to see if IACT could strengthen the effect of MMRP on self-management.

The medium size treatment effects on pain acceptance and pain willingness at post treatment and at post aftercare are in line with effect sizes found in two other comparisons of guided IACT and control condition for chronic pain patients [32,56]. Pain acceptance has been questioned as an outcome measure as it also possesses the features of a process measure and has been described as a moderator. A significant change in pain acceptance (CPAQ-8 short form) with medium effect size was however seen in association with other pain outcome measures in a network analysis, indicating that acceptance may be directly affected by rehabilitation and hence constitute an outcome measure by itself [57].

Psychological inflexibility has also been used as a moderator [17]. Although in this trial we measured psychological flexibility as a treatment outcome of the ACT interventions. The between group effect size on psychological inflexibility, fusion with pain and activity avoidance in the present trial were large at post aftercare. This is in line with the effect size found at post treatment in a trial where IACT was compared to WLC (d = 1.0) [17]. The present effect sizes are higher than what was found in another trial where IACT was compared to both an active control condition and WLC [58].

The treatment effect on affective distress at post treatment was moderate, in line with the effect size found at post treatment in a trial of IACT compared to control condition [32]. The treatment effect on pain specific self-efficacy in this trial was large at post aftercare and moderate at 1 year follow up. A trial of ICBT although compared to WLC also showed a large effect size at post treatment [59]. However, in a trial where ICBT was added to MMRP in primary care, no treatment effect was found on self-efficacy [22]. In addition, a trial of online pain management, although for a sample of older adults, did not yield a significant effect on PSEQ [60]. Improved self-efficacy at post treatment and at 1 year follow up in the present study, is promising. This could indicate that an IACT addition may enhance autonomy and improve patients’ perception of their ability to live well with pain.

When evaluating the treatment effects in the present study, one needs to bear in mind that the comparison condition, MMRP, included a substantial amount of rehabilitation interventions incorporating psychological interventions such as ACT [3]. Hence, it is not entirely applicable to compare effect sizes from this trial with effect sizes from WLC trials. In that light, it is promising to see that the effect of MMRP [61,62] can be strengthened with additional treatment effects on psychological outcomes when IACT is added.

However, this effect was not seen when ICBT with tenets of ACT, was added to MMRP in primary care [22]. One possible explanation is that pain-related psychological distress might be more prominent in a specialist clinic sample. A combination of psychological distress and disabling chronic pain is often a criterion for referral to specialist care. However, all specialist care patients do not experience pain-related psychological distress as their main concern [3]. Both pain duration and pain intensity last week in a sample of pain patients in primary care MMRP with an ICBT addition [23] was in line with the characteristics of participants in the present study. Possibly, the two samples do not differ substantially. Besides symptoms and level of care, we suggest that time since onset may explain why some benefit more than others from MMRP. Many agree that the best intervention for chronic pain is an early intervention that prevents chronicity [63]. Rather than differentiating between MMRP in primary and specialist care, it might be helpful to differentiate between interventions that prevent chronicity and interventions for living well with chronic pain.

The content and outline of IACT interventions in the present trial were adjusted to be congruent with and add on to the ACT sessions in MMRP. To make IACT and MMRP coherent, some elements of MMRP, as OT-, PT- and insomnia interventions, were embedded in the IACT addition. Although such a design may inflict on the comparison between MMRP and MMRP-IACT, this is an add-on comparison in an implementation context where coherence and synchronized treatments are essential. Nevertheless, ACT served as the overall rationale for interventions. Participants in internet-delivered interventions have previously experienced difficulties staying concentrated for a long time [64]. We therefore enriched the design with multimedia to improve attractiveness, ease learning and compensate for pain-induced cognitive deficits that may negatively affect adherence [65,66].

Two issues might have inflicted on the trial’s methodology. First, the website was designed and engineered for the present trial which left insufficient time for testing and stake-holder assessments prior to start. Secondly, different procedures for filling in forms, both digitally and with pen-and-paper, complicated data administration and made extraction and control of data challenging. The publication of the result was severely delayed, as it was more time consuming than anticipated to extract data. Overall, site-based, and digital interventions were easily integrated. However, reasons to decline participation and early-dropouts in the present study could indicate that the present IACT addition was too comprehensive. Committing to two simultaneous treatments might jeopardize engagement. However, patients’ needs differ. Some may appreciate an IACT addition for its accessibility, some may need guidance in home-based rehabilitation and yet others might use it to prepare for on-site sessions [67,68]. A conclusion from a previous study of ICBT added to MMRP was that adherence needs to be in focus [23], and that patients’ motivation and ability should be considered in treatment planning [22]. In addition, qualitative studies have drawn attention to the need of individualizing IACT to the needs and expectancies of chronic pain patients [69].

In conclusion, we found that individual internet-delivered ACT added to group-based MMRP may strengthen the effect on pain acceptance, psychological flexibility, pain-specific self-efficacy and reduce affective distress for patients in specialist care.

### 4.2. Strengths and Limitations

A majority of the participants in the MMRP-IACT group (57%, *n* = 28) were lost to post aftercare which is a major limitation. This is in line with the response rate in a large cohort study with an approximately 50 % drop-out rate at 1 year follow up [51]. However, the attrition rate also indicate that the aftercare intervention was not well received. One reason might be that the delivery mode differed from the main treatment, as it provided minimal therapist guidance and primarily served as a base for reminding, diary-keeping and evaluation of progress. Possibly, this setup did not match participants’ needs. Secondly, the aftercare intervention may have provided more of what had already been presented in the treatment phase in terms of content, rather than focus on participants’ needs at the time. Based on what is required for chronic pain patients to self-manage their condition after rehabilitation [70] a focus on activity engagement, problem solving, or behavioral activation could have been more beneficial. Noteworthy is however that the most treatments effects occurred during the aftercare intervention, at the same time as the largest drop-out occurred. This may indicate that aftercare is more needed by some and not fitted for all.

Considering the large drop-out in the present study we might conclude that a combination of the two full treatments may not be optimal. It has been suggested that IACT could target the psychological component of chronic pain more effectively than what is possible in a multimodal group based treatment [16]. The treatment effect on pain acceptance found in this trial, might indicate that IACT may be helpful before MMRP for those in need of psychological treatment outside the scope of MMRP. The effect on psychological flexibility and self-efficacy correspond well to the long-term target of ACT and could indicate an improved ability to self-manage chronic pain. IACT as aftercare following MMRP might enhance the effect of MMRP and provide guidance for those in need of additional support, as has been suggested before [10,71]. A third possibility is to combine IACT with MMRP to serve as home-based care for patients with mobility, geographical or financial restrictions, as has previously been found feasible [16]. Considering ageing populations and the expanding needs of pain rehabilitation, IACT could help make MMRP more accessible to these groups.

As pain patients seeking relief might be eager to accept any treatment available, a certain loss to follow-up is expected when they encounter treatment features. In this trial a relatively large group accepted participation without filling in pre- or post-measurements, also seen in another clinical trial of ICBT added to with MMRP [23]. The extensive drop out might, however, be regarded as a result in itself as it may reflect the clinical setting of and symptom panorama of chronic pain patients in specialist care. In addition, drop-out rates up to 60% are not unusual in trials with treatments focusing on behavior change [50]. However, attrition might also be related to implementation procedure. When IACT is being integrated, a clinic’s regular therapists might act both as e-therapists and on-site therapists. One potential implementation dilemma could be that patients may anticipate negative consequences if declining participation. Another risk is that patients might prefer reporting and problem solving in person rather than through the website, which could affect attractiveness of IACT and hence inflict engagement and adherence. A low adherence in the IACT addition may also be due to dissatisfaction with face-to-face MMRP [51]. Even though all patients in specialist pain care experience some psychological distress, everyone does not expect psychological interventions to improve their functioning. On the contrary, some are likely to expect pain relief from medical interventions [61]. The attractiveness of IACT is hence likely to also be affected by patients’ expectations of MMRP. It has to date been difficult to predict who might benefit from MMRP. However, an assessment of a patients’ initial expectations might indicate the appropriate combination of IACT added to MMRP [72,73].

The risk for Type II-error was increased in this trial as it was under-powered. We failed to recruit sufficient number of participants and had substantial loss of data. In addition, non-normal distribution of data, outliers and systematic non-random missing data made parametric testing less suitable and complicated use of mixed models and intention to treat analysis. Moreover, data would have been omitted using repeated measures. A limitation in this study, is therefore, the large amount of missing data which complicates the interpretation of the result. Last Observation Carried Forward could have been used to handle missing data, had it not been as extensive. Several outcomes and measurement points inflate the risk for Type 1 error. In addition, the study is biased as dropouts are not accounted for. However, all patients do not benefit from face-to face MMRP to begin with [51]. Furthermore, an uncontrolled trial of ICBT in tertiary pain care yielded significant improvements on several outcome measures for a subgroup of participants with clinical difficulties of disability [16]. However, in their overall sample, only measures of depression improved. Authors concluded that ICBT might be feasible for those pain patients who are unable or unwilling to attend tertiary MMRP on site due to clinical difficulties of disability [16]. We concur that a focus on subgroups of pain patients and how they receive, and experience internet-delivered interventions might pave way for recommendations on how to implement IACT in MMRP.

In this trial, cluster randomization was used not to inflict on the treatment integrity of MMRP by limiting the psychological benefits of group-based rehabilitation. This can be seen as a limitation. However, if patients in the same MMRP group would be randomized to different treatment conditions, they would not share the same treatment experiences, which would compromise group discussions and opportunities to benefit from each other’s reflections. Another way would have been to allocate participants to MMRP groups based on the randomization outcome. However, this would have disrupted clinical practice and been very difficult to motivate given the often complex social and financial situations in this patient group [20].

In spite of the above-mentioned limitations, the study has some strengths. It was set in a clinical service with already enrolled patients, with mean pain duration 7.2 years (SD 7.08). Patients enrolled in specialist care are likely to possess a more complex life situation, including occupational stressors, a large symptom panorama with long pain duration and also multimorbid diagnoses [1]. Participants in the present trial reported numerous prescribed medications, for pain, other physical and mental health conditions. This is expected in clinical trials of pain patients with typical multi- and comorbid conditions. In one example of a trial with IACT given to a tertiary care pain sample, patients with mental or physical illness or in a complicating life situation were not assessed as eligible for the study as their multimorbid conditions or life situation were hypothesized to negatively influence the uptake of IACT [18]. It may be that specialist care pain patients constitute a different sample compared to patients who self-recruit to IACT through adds in newspaper or on websites. Possibly they need interventions targeting a broader dimension of psychological distress, as depression, insomnia, worry and anxiety to fully comprehend the width of chronic pain. A lack of consideration of these debilitating comorbidities could help explain patients’ troubles to adhere. Another possibility is that a focus on experiential avoidance is helpful to pain patients with a demanding life situation, as it teaches helpful strategies to navigate emotions and compensate for executive dysfunction, without focusing on specific conditions.

## 5. Conclusions and Future Research

This study investigated the effect of adding IACT during and after MMRP for chronic pain patients in specialist care. The result show medium to large treatment effects in favor of the group with IACT added to MMRP on pain acceptance, psychological inflexibility, affective distress, and self-efficacy. We conclude that an IACT addition may enhance the effect of MMRP on psychological components. However, to date we do not know who needs, wants of will benefit from IACT added to MMRP. Internet-delivered interventions integrated in specialist pain care may also focus on physiotherapist interventions or return to work. To sum up, IACT added to MMRP raises new research questions.

First, internet interventions are rapidly disseminating in psychiatric, somatic and subclinical fields [11]. However, studies of implementation, process evaluations and benefit analysis from different stake holder perspectives are yet novel. One future possibility is IACT added to MMRP to compensate for low adherence in MMRP or to enable home-based rehabilitation, as has previously been suggested [16]. Another, is a step-wise approach where IACT follows face-to-face MMRP to prolong the effect of MMRP through aftercare, which has been found appreciated [10]. A third possibility is to offer IACT prior to MMRP to prepare patients for MMRP by giving individualized psychological treatment. If the latter would prove efficient, it might in the long run yield preventive interventions for broader samples to prevent high-risk persons to develop chronic pain [74]. We suggest that the next step of implementation research focus on IACT added before and after MMRP, to (1) treat psychological components that may lead to chronicity and (2) to prolong the effect of MMRP.

Second, qualitative studies have shown that pain patients’ experiences of IACT may vary substantially [69] and that patients’ expectations need to match treatment features to prevent attrition [72]. Since adverse events were not reported in the present trial, we cannot know if these affected adherence, outcome, or missing data. Negative effects of psychological pain treatments in general and internet-delivered pain interventions in particular are sparsely researched [75]. We suggest that further research on adverse events in both IACT and MMRP, might help patients, caregivers, and organizations to engage, adhere and be ready for this new combination of treatment delivery. 

## Figures and Tables

**Table 1:** Participants’ characteristics at enrollment.

		Intervention	Control
		(*n* = 49)	(*n* = 54)
Age, mean (SD)		36.35 (9.690)	35.93 (9.75)
Pain severity last week (VAS 0–10), mean (SD)		6.93 (1.83)	7.17 (1.45)
Years since pain onset, mean (SD)		8.4 (7.7)	5.9 (6.0)
	0–1 years, *n* (%)	9 (18.4)	7 (13)
	1–5 years	9 (18.4)	17 (31.5)
	5–10 years	6 (12.2)	9 (16.7)
	10–15 years	6 (12.2)	4 (7.4)
	15–20 years	5 (10.2)	2 (3.7)
	>20 years	3 (6.1)	2 (3.7)
Women, *n* (%)		43 (87.5)	45 (83.3)
Educational attainment, *n* (%)			
	Elementary (1–9 years)	4 (8.2)	5 (9.3)
	Secondary (10–12 years)	28 (57.1)	32 (59.3)
	University (>12 years)	13 (26.5)	11 (20.4)
	Other	1 (2.0)	0 (0)
	Unknown	3 (6.1)	6 (11.1)
Working condition at enrollment, *n* (%)			
	Permanent or self–employed	35 (71.4)	28 (51.9)
	Temporary employment	4 (8.2)	2 (3.7)
	Unemployed	9 (18.4)	14 (25.9)
	Student	2 (4.1)	1 (1.9)
	Outside the labor market	0 (0.0)	4 (7.4)
	Working full time	14 (28.6)	10 (18.5)
	Working to any degree	36 (73.5)	32 (59.3)
Sickness benefits to any degree, *n* (%)		19 (38.8)	19 (35.2)

**Table 2:** Means (SD) and medians (Md) on outcome measures Chronic Pain Acceptance Questionnaire (CPAQ), Psychological Inflexibility in Pain Scale (PIPS), Pain Self-efficacy Questionnaire (PSEQ) and Multidimensional Pain Inventory (MPI), for MMRP-IACT and MMRP respectively at pretreatment, at post treatment, at post aftercare intervention and at 1 year follow up.

	Group	Pretreatment			Post Treatment			Post Aftercare			1 Year Follow-Up		
		*n*	Mean (SD)	Md	*n*	Mean (SD)	Md	*n*	Mean (SD)	Md	*n*	Mean (SD)	Md
CPAQ													
Pain willingness	MMRP-IACT	49	23.6 (8.0)	23.0	39	29.1 (7.4)	29.0	19	33.3 (7.9)	35.0	27	30.0 (8.7)	30.0
	MMRP	54	21.7 (8.0)	21.0	37	25.2 (7.3)	24.0	30	28.7 (7.1)	28.5	30	28.80 (9.0)	27.0
Activity engagement	MMRP-IACT	49	26.1 (12.6)	25.0	38	35.6 (12.6)	36.5	19	39.8 (14.1)	39.0	25	36.5 (13.1)	37.0
	MMRP	54	25.6 (9.9)	24.5	37	32.3 (10.1)	31.0	30	34.1 (10.5)	33.5	28	33.5 (13.4)	31.0
Total	MMRP-IACT	49	49.7 (18.6)	49.0	37	64.8 (17.0)	67.0	19	73.1 (20.0)	77.0	25	67.0 (19.2)	64.0
	MMRP	54	47.3 (14.4)	45.0	37	57.5 (15.9)	56.0	30	62.8 (16.0)	59.5	28	62.6 (21.4)	59.0
PIPS													
Avoidance	MMRP-IACT	49	35.8 (11.5)	35.0	39	30.0 (10.3)	29.0	21	24.4 (9.0)	23.0	18	26.6 (10.2)	26.5
	MMRP	54	38.0 (8.9)	38.0	38	31.5 (8.4)	30.5	30	30.7 (9.9)	31.5	22	30.3 (8.7)	30.5
Fusion	MMRP-IACT	49	20.4 (5.0)	21.0	39	17.0 (5.4)	18.0	21	14.4 (4.7)	16.0	18	15.5 (3.5)	15.5
	MMRP	54	22.0 (4.2)	22.0	38	18.5 (4.1)	18.0	30	17.6 (4.7)	18.5	22	17.7 (4.4)	19.0
Total	MMRP-IACT	49	56.3 (14.4)	55.0	39	46.9 (13.4)	48.0	21	38.8 (11.9)	36.0	18	42.1 (12.3)	42.5
	MMRP	54	60.1 (11.7)	59.5	38	50.0 (11.4)	49.5	30	48.3 (13.2)	51.0	22	48.0 (12.0)	50.5
PSEQ													
	MMRP-IACT	48	26.8 (12.9)	26.5	38	36.2 (13.5)	36.0	21	40.5 (11.1)	42.0	18	38.9 (13.2)	41.5
	MMRP	54	25.7 (11.9)	23.0	38	30.8 (11.2)	30.5	30	31.0 (13.5)	31.0	22	29.8 (13.2)	31.5
MPI													
Pain severity	MMRP-IACT	46	4.1 (1.1)	4.0	42	4.0 (1.2)	4.3	16	3.6 (0.7)	3.5	27	4.0 (1.2)	4.0
	MMRP	52	3.9 (1.0)	4.0	37	4.2 (1.1)	4.3	29	3.5 (1.2)	3.5	30	3.7 (0.9)	3.8
Pain interference	MMRP-IACT	46	4.0 (1.1)	3.9	42	4.1 (0.9)	4.1	16	3.1 (1.2)	3.3	27	3.8 (1.1)	3.9
	MMRP	52	4.0 (1.0)	4.1	37	4.2 (1.0)	4.3	29	3.5 (1.4)	3.9	30	4.0 (1.2)	4.3
Life control	MMRP-IACT	46	2.6 (1.1)	2.8	41	3.3 (1.2)	3.3	16	3.5 (0.8)	3.5	27	2.9 (1.4)	2.5
	MMRP	52	2.9 (0.8)	3.0	37	2.8 (0.9)	2.8	29	3.1 (1.1)	3.3	30	3.1 (1.2)	3.0
Affective distress	MMRP-IACT	46	3.3 (1.4)	3.0	41	2.9 (1.3)	2.7	16	2.9 (1.6)	2.8	27	3.6 (1.3)	3.7
	MMRP	52	3.4 (1.0)	3.3	37	3.5 (1.1)	3.3	29	2.9 (1.1)	3.0	30	3.2 (1.5)	3.0
Social support	MMRP-IACT	46	4.1 (1.4)	4.5	41	3.7 (1.2)	4.0	16	3.5 (1.7)	3.8	27	3.7 (1.1)	3.7
	MMRP	52	3.9 (1.4)	4.0	37	3.8 (1.3)	4.0	29	3.5 (1.7)	3.5	30	3.4 (1.4)	3.7

## Data Availability

The data presented in this study may be obtained by the corresponding author upon reasonable request.

## Supplementary Materials

The following are available online at https://www.mdpi.com/article/10.3390/jcm10245872/s1, Table S1: Outline MMRP, Table S2: Outline IACT added during MMRP, Table S3: Outline IACT added after MMRP.
