# Supplementary material for: Internet-Delivered Acceptance and Commitment Therapy Added to Multimodal Pain Rehabilitation: A Cluster Randomized Controlled Trial
Source: J Clin Med. 2021 Dec 14;10(24):5872. doi: 10.3390/jcm10245872 (PMC8705416; doi:10.3390/jcm10245872)
Supplement: Supplementary file 1 [file jcm-10-05872-s001.zip › Supplementary nr Table S1 outline MMRP.pdf]

Supplementary Material Table S1

Table S1. Outline of content in MMRP [1]. Interventions in the ACT-sessions are derived from or modified from ACT-literature [2] [3-5].

Week	Educational interventions	ACT	OT	PT	Insomnia intervention	Homework
Pre	Rationale. Expectations for MMRP. Motivational exercise. Goal-setting.					Ongoing LP. Sleep registration. Goal-setting.
1	Goal-setting. Values intervention. Behavior change. Biology, functions and mechanisms of chronic pain. Mindfulness.	Rationale. Group dynamics intervention. Values interventions: Alice and the Cheshire cat; The Diamond; Differences between goal/values/result. Mindfulness exercises (notice 3 things). Introducing creative hopelessness; Man in a hole. Serenity prayer (short version).	Inventory and discussion of activity patterns and – execution and meaningful activities (COPM) and time distribution. Return to work.	LA: Experiences of physical training. BBA: Stabilizing exercises. Present moment. CT: Stabilizing.	Group dynamics intervention. Goals and expectation. Rationale for sleep restriction. Biology and function of sleep and the effect of sleep medication. Evaluation of sleep registration.	Goals and expectations. Life-compass. Activity diary with reflections. Evaluating LP. BBA-exercise. Sleep registration and educational text.
2	Behavior regulation and common psychological reactions to pain.	Values: A long journey. Creative hopelessness. Mindfulness: Leaves on a stream. Defusion interventions: Feed the tiger, Tug-of-War with a monster. BEVS [6].	Analysis of activity pattern, problem solving and inventory of possible alternative strategies. Skills training in activity (EMG-biofeedback). Introducing pacing,	BBA: Stabilizing exercises. Present moment. CT: Motor control	Sleep restriction, stimulus control. Behavior change techniques. Evaluating sleep diary.	Observing inner sensations. Hindrances in Life compass. Behavioral experiment alternative strategy in meaningful activity (OT). Exercise diary of LP. BBA exercise. Sleep diary. Apply stimulus control.

			graded activity and present moment in activity.			
3	Anxiety psychoeducation	Values and mindfulness interventions (breathing). Introducing acceptance (Take your keys with you) and committed actions. The shark trap.	Skills training in everyday life. Focus on meaningfulness in work/studies. Behavioral activation intervention. Occupational role and significance. WRI assessment	LA: Joy of movement<br>BBA: Stabilizing exercises with present moment.<br>CT: Graded exercise.	Educational intervention: Biology and function of sleep. Evaluating sleep diary.	CA worksheet; BEVS. Reflect on significance of work. Exercise diary of LP. BBA exercise.<br>Sleep diary. Learning by doing- exercise.
4		Hindrances. Introducing perspective/self as context (Content on cards, Observer exercise). Acceptance as an alternative to control (Passengers on the bus). Mindfulness (Breathing). Committed actions.	Analysis of activity pattern, problem solving and inventory of possible alternative strategies. Homework evaluation. Assessment of work situation; strengths and weaknesses. My skills and competence.	BW: physical activity pyramid<br>BBA: Centering, breathing, present moment<br>CT: pacing in exercise	Visualizing based relaxation exercises. Evaluating sleep diary.	CA worksheet. Plan for changes in work life and home setting to reach a sustainable work situation. Ongoing behavioral activation. Exercise diary of LP. BBA exercise. Sleep diary. Practice visualization.
5		Acceptance (Bubble in the road, Jump Exercise, Tin Can Monster, Joe the Bum). Mindfulness skills. CA,	Skills training in everyday life (EMG, video	BW: Goals and valued direction.	Strategies for worry. Soldiers in the parade. Evaluating sleep diary	Committed actions. Exercise diary of LP. BBA exercise. Sleep diary. Test

		long-term change (chessboard, serenity prayer)	feedback). Problem solving and planning for return to work. Continued behavioral activation. WRI feedback. Plan meeting with social insurance representative	BBA: Centering, breathing, present moment<br>CT: Autopilot, workability		techniques to handle worry and rumination. Prepare for aftercare plan.
6	Summary and evaluation. Aftercare plan. Letter to myself. Rational for long-lasting behavioral change, ongoing mindfulness training and continuous behavioral activation.	Aftercare. Pain exposure (Physicalizing). Educational intervention: Act/React. Mindfulness.	Skills training follow-up. Behavioral activation follow-up. Feedback on skills training (video). Present moment exercise: want/must. Summarizing. COPM measurement.	BW: Maintenance, strategies for hindrances.<br>BBA: Centering, Breathing, present moment<br>CT: Maintenance. Tippa test.	Aftercare plan with maintenance strategies. Problem solving. Sleep hygiene. Evaluating sleep diary.	Evaluating exercise diary LP. Maintenance plan for physical exercise and BBA. CT exercises.

Abbreviations: MMRP: multimodal rehabilitation program; ACT: Acceptance and commitment therapy; OT: occupational therapy; PT: physical therapy; LP: Low-intensive physical activity; COPM: Canadian occupational performance measure; LA: Light aerobics; BBA: Basic Body awareness; CT: Circuit training; BEVS: Bull's eye Values Survey; EMG: Electromyogram; WRI; CA: Committed action; Worker Role Inventory; BW: beginner workout.

References

1. Gerdle B, Molander P, Stenberg G, Stålnacke BM, Enthoven P. Weak outcome predictors of multimodal rehabilitation at one-year follow-up in patients with chronic pain - a practice based evidence study from two SQRP centres. BMC Musculoskeletal Disorders. 2016;17(1):1-14.

2. Buhrman M, Skoglund A, Husell J, Bergström K, Gordh T, Hursti T, et al. Guided internet-delivered acceptance and commitment therapy for chronic pain patients: A randomized controlled trial. Behaviour Research and Therapy. 2013;51(6):307-15.
3. Robinson P, Wicksell RK, Olsson GL. ACT with Chronic Pain Patients. In: Hayes SC, Strosahl KD, editors. A practical guide to Acceptance and Commitment Therapy. New York: Springer; 2004.
4. Hayes SC, Strosahl KD, Wilson KG. Acceptance and Commitment Therapy. An experiential approach to behavior change: Guilford Publications; 1999.
5. McCracken LM. Contextual cognitive-behavioral therapy for chronic pain. International Association for the Study of Pain, editor: Lippincott, Williams and Wilkins.; 2005.
6. Lundgren T, Luoma JB, Dahl J, Strosahl K, Melin L. The Bull's-Eye values survey: A psychometric evaluation. Cognitive and Behavioral Practice. 2012;19(4):518-26.
