# Supplementary material for: Internet-Delivered Acceptance and Commitment Therapy Added to Multimodal Pain Rehabilitation: A Cluster Randomized Controlled Trial
Source: J Clin Med. 2021 Dec 14;10(24):5872. doi: 10.3390/jcm10245872 (PMC8705416; doi:10.3390/jcm10245872)
Supplement: Supplementary file 1 [file jcm-10-05872-s001.zip › Supplementary nr Table S2 outline IACT added during MMRP.pdf]

Supplementary material Table S2

Table S2. Outline of content in IACT added during MMRP. Some exercises are designed for this trial, although derived from or modified from ACT-literature [1] [2-4].

Week	Experiential exercise (text and audio track)	Mindfulness (text and audio track)	Weekly update/ Educational text	Physical exercise	Evaluation (BEVS) [5]	Body awareness (audio tracks)
Pre	"Man in the Hole" and "Creative hopelessness"-work sheet	Educational text. Exercise "Breathing"	Educational text on IACT and MMRP. Measurements and Informed consent			
1	Rationale for ACT	"Staying present"	Goal setting	Educational text and goal setting		
2	"A long journey"	"Exploring"	Information from MMRP and lecture on pain mechanisms	Exercise diary	Educational text on BEVS and "Life values"-work sheet	"Body scan lying down"
3	"The shark trap"	"The surroundings"	Encouraging goal evaluation	Exercise diary	Committed actions and evaluation	"Balancing sitting" and "Meditation"
4	"Chessboard"	"Thoughts"	Exercise routines. Meeting with social insurance representatives	Exercise diary	Committed actions and evaluation	"Balancing standing" and standing exercises
5	"Passengers on the bus"	Educational text. "Practicing willingness"	Summary of MMRP	Exercise diary	Committed actions and evaluation	"Behavioral based relaxation"
6	"Fare-well party"	"The scientist"	Aftercare. Measurements	Exercise diary	Committed actions and evaluation	

Abbreviations: MMRP: Multimodal rehabilitation program; IACT: Internet-delivered acceptance and commitment therapy; BEVS: Bull's eye Values Survey.

References

1. Buhrman M, Skoglund A, Husell J, Bergström K, Gordh T, Hursti T, et al. Guided internet-delivered acceptance and commitment therapy for chronic pain patients: A randomized controlled trial. Behaviour Research and Therapy. 2013;51(6):307-15.
2. Robinson P, Wicksell RK, Olsson GL. ACT with Chronic Pain Patients. In: Hayes SC, Strosahl KD, editors. A practical guide to Acceptance and Commitment Therapy. New York: Springer; 2004.
3. Hayes SC, Strosahl KD, Wilson KG. Acceptance and Commitment Therapy. An experiential approach to behavior change: Guilford Publications; 1999.

4. McCracken LM. Contextual cognitive-behavioral therapy for chronic pain. International Association for the Study of Pain, editor: Lippincott, Williams and Wilkins.; 2005.
5. Lundgren T, Luoma JB, Dahl J, Strosahl K, Melin L. The Bull's-Eye values survey: A psychometric evaluation. Cognitive and Behavioral Practice. 2012;19(4):518-26.
