# Supplementary material for: Internet-Delivered Acceptance and Commitment Therapy Added to Multimodal Pain Rehabilitation: A Cluster Randomized Controlled Trial
Source: J Clin Med. 2021 Dec 14;10(24):5872. doi: 10.3390/jcm10245872 (PMC8705416; doi:10.3390/jcm10245872)
Supplement: Supplementary file 1 [file jcm-10-05872-s001.zip › Supplementary nr Table S3 outline IACT added after MMRP.pdf]

Supplementary material Table S3

Table S3. Outline of content in MMR-IACT Aftercare. Some exercises are designed for this trial, although derived from or modified from ACT-literature [1] [2] [3] [4].

Week	Educational texts	Committed actions (BEVS) [5]	ACT educational texts	Experiential exercises (text and audio track)	Occupational therapy educational texts (flexible starting week)	Body awareness and relaxation	Physical therapy exercises	Sleep educational texts
AC 1	Rationale: Aftercare	Worksheet: Values and committed actions	Valued living	"The Cheshire cat", "Skiing" and "The diamond"			Exercise diary	
AC 2		Weekly plan and BEVS	Rationale: ACT		Rationale: OT and aftercare	Educational text: Physical therapy	Exercise diary	Rationale: Sleep and aftercare
AC 3	SMART goals	Weekly plan and BEVS	Hindrances. Anxiety reactions.	"Two scales"	Learning by doing	Educational text: Body awareness	Educational text on physical exercises. Exercise diary	Introduction
AC 4	Activity planning	Weekly plan and BEVS	Belly breathing and hyperventilation.	Mindfulness: "Breathing exercise"	Balance in and between activities	Basic exercises in body awareness	Educational text on exercise diary. Exercise diary	Sleep restriction
AC 5	Problem-solving	Weekly plan and BEVS	Dysfunctional habits and alternatives.	"Leaves on a stream"	Sleep	Educational text: Relaxation	Circuit training. Music (Audio tracks). Exercise diary	Biology and function of sleep
AC 6	Communication	Weekly plan and BEVS	Perspective on thoughts	"Observer exercise"	Work life or studying	Continuous exercises in body awareness	Exercise diary	Visualization with exercise (audio track)
AC 7		Weekly plan and BEVS	Ways to practice willingness.	"The watchdog"		References for further training	Exercise diary	Worry
AC 8		Weekly plan and BEVS	Behavioral activation.	"Joe the Bum"			Exercise diary	Sleep hygiene, problem

				solving and maintenance
AC 9	Summary of change	Weekly plan and BEVS	Exercise diary	
AC 10	Summary of steps taken and evaluation			
Post AC	Plan for continuous aftercare. Post measurements			

Abbreviations: MMRP: multimodal rehabilitation program; IACT: Internet-delivered acceptance and commitment therapy; AC: Aftercare; BEVS: Bull’s eye Values Survey.

References

1. Buhrman M, Skoglund A, Husell J, Bergström K, Gordh T, Hursti T, et al. Guided internet-delivered acceptance and commitment therapy for chronic pain patients: A randomized controlled trial. Behaviour Research and Therapy. 2013;51(6):307-15.
2. Robinson P, Wicksell RK, Olsson GL. ACT with Chronic Pain Patients. In: Hayes SC, Strosahl KD, editors. A practical guide to Acceptance and Commitment Therapy. New York: Springer; 2004.
3. Hayes SC, Strosahl KD, Wilson KG. Acceptance and Commitment Therapy. An experiential approach to behavior change: Guilford Publications; 1999.
4. McCracken LM. Contextual cognitive-behavioral therapy for chronic pain. International Association for the Study of Pain, editor: Lippincott, Williams and Wilkins.; 2005.
5. Lundgren T, Luoma JB, Dahl J, Strosahl K, Melin L. The Bull's-Eye values survey: A psychometric evaluation. Cognitive and Behavioral Practice. 2012;19(4):518-26.
